# Instructional multimedia: An investigation of student and instructor attitudes and student study behavior

**DOI:** 10.1186/1472-6920-11-38

**Published:** 2011-06-21

**Authors:** A Russell Smith, Cathy Cavanaugh, W Allen Moore

**Affiliations:** 1 Lynchburg College, Doctor of Physical Therapy Program, Lynchburg, Virginia 24501, USA; 2 University of Florida, School of Teaching and Learning, Gainesville, Florida 32611, USA

## Abstract

**Background:**

Educators in allied health and medical education programs utilize instructional multimedia to facilitate psychomotor skill acquisition in students. This study examines the effects of instructional multimedia on student and instructor attitudes and student study behavior.

**Methods:**

Subjects consisted of 45 student physical therapists from two universities. Two skill sets were taught during the course of the study. Skill set one consisted of knee examination techniques and skill set two consisted of ankle/foot examination techniques. For each skill set, subjects were randomly assigned to either a control group or an experimental group. The control group was taught with live demonstration of the examination skills, while the experimental group was taught using multimedia. A cross-over design was utilized so that subjects in the control group for skill set one served as the experimental group for skill set two, and vice versa. During the last week of the study, students and instructors completed written questionnaires to assess attitude toward teaching methods, and students answered questions regarding study behavior.

**Results:**

There were no differences between the two instructional groups in attitudes, but students in the experimental group for skill set two reported greater study time alone compared to other groups.

**Conclusions:**

Multimedia provides an efficient method to teach psychomotor skills to students entering the health professions. Both students and instructors identified advantages and disadvantages for both instructional techniques. Reponses relative to instructional multimedia emphasized efficiency, processing level, autonomy, and detail of instruction compared to live presentation. Students and instructors identified conflicting views of instructional detail and control of the content.

## Background

In order to meet the educational needs of a diverse population of students, physical therapist educators are utilizing instructional multimedia to teach psychomotor skills [1-3]. Traditional strategies to teach psychomotor skills in healthcare education include lecture, textbooks, self-instruction, and live demonstration [2]. Instructional multimedia has been applied as a component of classroom activities, in pre-class preparation, or as a stand-alone learning experience [4-6]. Increasing integration of instructional multimedia across disciplines has been noted as access to educational technology has increased [7]. Some recently employed multimedia technologies, including video, film, DVD/CD-ROM, computer simulations, slide presentations, audio recordings, and web based content, have been utilized to present lectures, supplement classroom activities, and demonstrate psychomotor skills [1-3,8-11]. Advantages of instructional multimedia include increased availability and repetition of instructional content, improved ability of students to learn at their own pace, increased student control of material, less demand on instructor time, and the provision of an alternative approach to describe complex topics or three-dimensional relationships. Instructional multimedia may help instructors address a range of diverse student needs including increasing study time and addressing multiple learning preferences [3,5-7,12,13]. These advantages, especially when applied in a professional degree program, align well with research on development of expertise, which attributes such development to intense practice with directed feedback on performance [14]. Given that physical therapy is to a large extent a psychomotor domain of practice, a physical therapy degree program may be considered in a sense predominantly a performance improvement program [15]. Thus, the instructional multimedia program described here was designed to improve student performance through practice and feedback without increasing lab, classroom, or instructor time.

Much of the health education research concerning instructional multimedia has focused on cognitive [16,17] and/or psychomotor [4,16,18-20] performance. Recently, investigations concerning student/instructor perceptions of instructional multimedia have been published [21,22]. However, published reports examining student/instructor perceptions of instructional multimedia in physical therapy education were not found. Where cognitive and psychomotor performance are concerned, traditional forms of instruction and teaching with instructional multimedia yield similar levels of performance among students and increased efficiency in learning time [19]. For example, Kinney *et al. *(1997) found no differences in written test scores between physical therapist students receiving instructional multimedia and students receiving interactive lecture presentation for the management of patients with carpal tunnel syndrome. However, the authors reported less time was needed for the multimedia instruction group (mean 82.6 minutes) to complete the lesson compared to the interactive lecture group (124.6 minutes) [19]. Barker (1988) investigated the effectiveness of interactive videodiscs in the acquisition of upper extremity range of motion assessment skills in 40 undergraduate physical therapist students. Although no differences in written or psychomotor performance were observed between the multimedia group and the traditional group, participants in the multimedia group reported greater time practicing than the traditional group, 11.16 hours as compared to 8.39 hours [4].

In health science education, when instructional multimedia increases the richness of the experience and are accompanied by guidance from instructors or professionals, performance gains may be seen [15], as in Holzinger et al.'s study of a supported simulation to teach blood flow. The study showed increased knowledge when video, computer-based simulation and learning guidance were combined to scaffold learning through increasingly complex stages. In fact, the more complex the learning demands, the greater the contribution of dynamic media may be to the learning process [23].

Instructional multimedia for cognitive and psychomotor learning across higher education appears to be most effective as a complement to classroom instruction rather than a substitute for classroom instruction [22,24]. Previous investigations of nursing students' attitudes towards instructional multimedia found learners identified that instructional multimedia enhanced learning, allowed for greater flexibility, and provided a platform for independent self-management of learning [22,25,26]. While students appreciate the increase in control instructional multimedia provides them over their learning, many students may find motivation to be a challenge in the absence of instructors or other students. In addition, learners may have preexisting attitudes about the value of instructional multimedia based on their past learning experiences and knowledge of their personal learning preferences. In fact, students' attitudes toward the instructional multimedia format are significant predictors of course performance [27]. For example, students who believe they have difficulty learning from reading will place high value on image-rich multimedia, while students who state they learn best through interpersonal interaction will place low value on the time they spend using instructional multimedia [28].

The degree of control students are able to exercise in a course activity depends on the structure and flexibility designed into the activity by the instructor. Instructor satisfaction derives from congruence between the instructor's beliefs about learning and the methods the instructor uses to facilitate learning [29]. Instructor motivation to use instructional multimedia is based on the premise that students in health professions have diverse needs and a preference for multimodal learning tools [30-32], and that multimedia may better serve multiple learning styles [27].

Students/subjects in the present study were provided video clips with concurrent text of psychomotor techniques (CD-ROM) to review and practice prior to the classroom meeting. This methodology is similar to learning in an online environment where students learn through interaction within the online environment, not during actual physical interaction with an instructor. In this way, the instruction represents a blended course model and provides insight into a blended physical therapy course design. The application of video demonstrations with audio narration and text for learning numerous psychomotor skills is supported by Mayer's multimedia learning principle of modality [33]. Students using the physical therapy multimedia materials acquired knowledge of the techniques through simultaneous visual and auditory modes, increasing the likelihood that the students would successfully apply the techniques in the lab and clinic setting.

Bates and Bartolic-Zlomislic (1999) proposed the following as a framework to investigate the effects of independent learning in an online environment: 1) performance-driven benefits (e.g., learning outcomes, student satisfaction, instructor satisfaction); 2) value-driven benefits (e.g., access, flexibility, and ease of use); and 3) value-added benefits (e.g., increased revenue related to new product/service generated, and reduced traffic/parking needs) [34]. The focus of investigations of instructional multimedia in physical therapist education has often been limited to the effect on learning outcomes [1-3]. The present study focuses on the first two benefits as presented by Bates and Bartolic-Zlomislic, specifically performance-driven and value driven benefits.

The performance-driven benefit of student satisfaction is of particular importance because of its task, course, and program level implications. Students who are satisfied with their own performance and with their progress are more likely to persist in their educational endeavors [35]. Therefore, it is in the best interests of the student, the instructor, and the program for students to reach a motivating level of satisfaction in their courses. In the case of the physical therapy courses investigated in this study, students were asked to use instructional multimedia for self-directed learning, a method for which students vary in readiness and need for support [36]. The continual need for learning within individuals and the need to explore and to develop responsibility among maturing learners [37] may be satisfied with instructional multimedia tools that students can control and use as their abilities dictate.

The purpose of this study was to examine the effects of instructional multimedia on student study behavior, and student and teacher attitudes toward instructional multimedia. Two research questions guided this study: (1) What are the experiences of students and teachers of instructional multimedia as an instructional strategy? and (2) Does instructional multimedia affect student study behavior?

## Methods

### Sample

Students from two entry-level physical therapist graduate education programs agreed to participate in the study. Program 1 had 27 participants (18 females and nine males, mean age = 24.69 years) in a first-semester course. Program 2 had 23 participants (13 females and 10 males, mean age = 25.13) in a second-semester course. Both groups were enrolled in introductory level orthopedic physical therapy courses.

### Procedures

The study was conducted over five weeks at each of the two universities. During the first week of the study, all participants provided written informed consent and basic demographic information. Next, participants from each university were divided into one of two groups, Group A or Group B. Students in Group A received reading assignments and content objectives related to knee special tests and accessory movement testing. Participants in Group B received compact discs (CDs) with knee techniques, in addition to, the same reading assignments and content objectives that Group A received. During week two of the study, students from Group A received live instructor demonstration of knee techniques followed by practice with instructor feedback. Participants in Group B, having received CDs during week one of the study, moved directly to practice with instructor feedback. Both groups received a total of 120 minutes of time with the instructor during separate sessions. Group A's session included live demonstration, Group B's session did not include live demonstration. During week three of the study, participants received instruction on ankle/foot techniques. During this week, Group A received CDs and practice time with instructor feedback, and Group B received live demonstration and practice time with instructor feedback. Once again, each group received a total of 120 minutes with the instructor during separate sessions. During weeks 4 and 5 of the study, students underwent practical and written testing related to knee and ankle/foot techniques and students and instructors completed the questionnaire examining their attitudes toward the learning process. Data pertaining to performance on written and practical examinations were published previously [3].

### Research Design

The independent variable was instructional method, either multimedia instruction or traditional classroom demonstration. The dependent variables were student study behavior, and student and instructor attitudes. Student and instructor attitudes and student study behavior were assessed by a written questionnaire consisting of seven statements with Likert scales and six open-ended questions. Approval for this study was obtained from the Institutional Review Board of the University of North Florida.

### Instructional Multimedia

**Figure 1:**
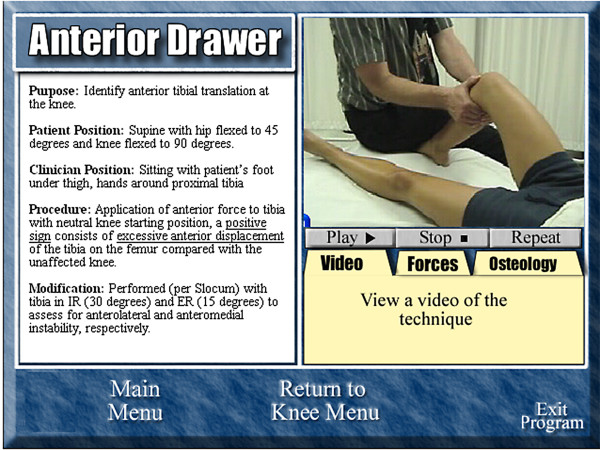
**Screen capture of interactive video layout**. Study participants were given a CD-ROM containing multiple short videos of various ankle and knee examination skills to practice before class.

Instructional CDs containing psychomotor skills of accessory movement testing and special tests for examination of the knee or ankle/foot were developed by the primary investigator. Twenty-three techniques were presented for the knee and 20 techniques were presented for the ankle/foot. Each technique was described in text format with concurrent audio-video presentation of the technique (Figure 1). The audio/video presentation of each of the skills was approximately 30 seconds in length. Media was rendered into a Flash^® ^format and students were required to install Adobe's^® ^free Flash Player^® ^to view the videos on their personal computers.

### Tests and Instrumentation

The written questionnaire used in this study to assess students' attitudes toward each instructional strategy was modified from a survey developed by Toth-Cohen (1995) [38] (Appendix 1). Instructors' reactions to the instructional strategy were assessed by a written questionnaire similar to the student version (Appendix 2). Student study time alone and with classmates were recorded by the student on the written questionnaire. Total study time was calculated by adding the reported alone study time and the classmate study time.

### Data Analysis

Independent, group, and total study time data were analyzed using ANOVA. Analysis included a comparison of study times between the two physical therapy programs and between the two instructional methods. Additionally, descriptive analysis was conducted for study times for each program and for the instructional strategies. For study time analysis, data were transferred to SPSS 18 and alpha level was set at p < .05. Qualitative descriptive analysis was performed for the Likert scale statements and for the open-ended questions. In addition, response frequency for each of the Likert statements were tabulated using Microsoft Excel 2007. Lastly, Mann-Whitney tests were performed for each of the seven Likert statements within the knee and ankle groups. Data was transferred to SPSS 18 for this analysis and alpha level was set at p < .05.

## Results

### Study Times

Twenty-five subjects in the live demonstration knee group completed the questionnaire regarding study times. The mean time studying alone was 47 minutes, mean group study time was 24.8 minutes, and total study time for this group was 71.8 minutes. Twenty-one students in the knee instructional multimedia group completed the questionnaire regarding study time. The mean study alone time for this group was 32.9 minutes, group study time mean was 32.9 minutes and total study time was 65.7 minutes. Twenty-three subjects in the live demonstration ankle group completed the questionnaire regarding study times. The mean study alone time was 30.7 minutes, mean group study time was 32.6 minutes, and total study time for this group was 63.3 minutes. Twenty students in the ankle instructional multimedia group completed the questionnaire regarding study time. The mean study alone time for this group was 24.3 minutes, group study time mean was 7.5 minutes and total study time was 31.8 minutes (Table 1). ANOVAs comparing study times for each instructional method and for each program are presented in Table 2. Results of ANOVA related to student study behavior related to knee content indicated no main effects and no interaction. Results for study alone and total study time of the ankle content indicated no interaction between strategy and school and no significant main effects. Results of ANOVA related to study time with classmates of ankle content indicated there was no interaction between strategy and school, however, group study time was significantly different when comparing instructional strategies and group study time (F = 6.496; p = 0.015). Students in the live ankle demonstration group spent more time studying in groups than students in the instructional multimedia group.

**Table 1:** Descriptive statistics for knee and ankle study times

			Knee			Ankle		
**Program**	**Instructional Method**		**Alone Study Time**	**Group Study Time**	**Total Study Time**	**Alone Study Time**	**Group Study Time**	**Total Study Time**

#1	Live Demo	N	14	14	14	12	12	12
		Mean (minutes)	44.6	11.8	56.4	27.5	25	52.5
		SD	42.2	32.3	63	36.5	41.8	67.7
	
	CD Demo	N	11	11	11	11	11	11
		Mean (minutes)	19.5	36.8	56.4	18.2	0	18.2
		SD	25.5	50.7	69.4	22.2	0	22.2
	
	Total	N	25	25	25	23	23	23
		Mean (minutes)	33.6	22.8	56.4	23	13	36.1
		SD	37.4	42.4	64.5	30.2	32.2	53.1

#2	Live Demo	N	11	11	11	11	11	11
		Mean (minutes)	50	41.4	91.4	34.1	40.9	75
		SD	32.6	34.9	52.4	25.6	40.9	53
	
	CD Demo	N	10	10	10	9	9	9
		Mean (minutes)	47.5	28.5	76	31.7	16.7	47.2
		SD	19.3	36	40.1	40.3	18.5	52.7
	
	Total	N	21	21	21	20	20	20
		Mean (minutes)	48.8	35.2	84	33	30	63
		SD	26.5	35.2	46.5	32.1	34.3	53.3

Programs Combined	Live Demo	N	25	25	25	23	23	23
		Mean (minutes)	47	24.8	71.8	30.7	32.6	63.3
		SD	37.6	36	60.1	31.2	41.2	60.8
	
	CD Demo	N	21	21	21	20	20	20
		Mean (minutes)	32.9	32.9	65.8	24.3	7.5	31.8
		SD	26.4	43.4	56.9	31.5	14.7	40.6
	
	Total	N	46	46	46	43	43	43
		Mean (minutes)	40.5	28.5	69	27.7	20.9	48.6
		SD	33.4	39.3	58.1	31.1	33.9	54.2

**Table 2:** Results of ANOVA comparing study times between subjects and program, and subjects and instructional method

		Knee			Ankle		
		
		df	F	Significance	df	F	Significance
Study Alone	Program	1	3.025	.089	1	1.069	.308
	Instructional Strategy	1	2.077	.157	1	.366	.549
	Program X Instructional Strategy	1	1.392	.245	1	.126	.724

Group Study	Program	1	.851	.362	1	2.843	.100
	Instructional Strategy	1	.279	.600	1	6.469	.015*
	Program X Instructional Strategy	1	2.704	.108	1	.002	.969

Total Study	Program	1	2.496	.122	1	2.609	.114
	Instructional Strategy	1	.199	.657	1	3.787	.059
	Program X Instructional Strategy	1	.196	.660	1	0.42	.839

*Significance ≤ .05.

### Student Attitudes

**Figure 2:**
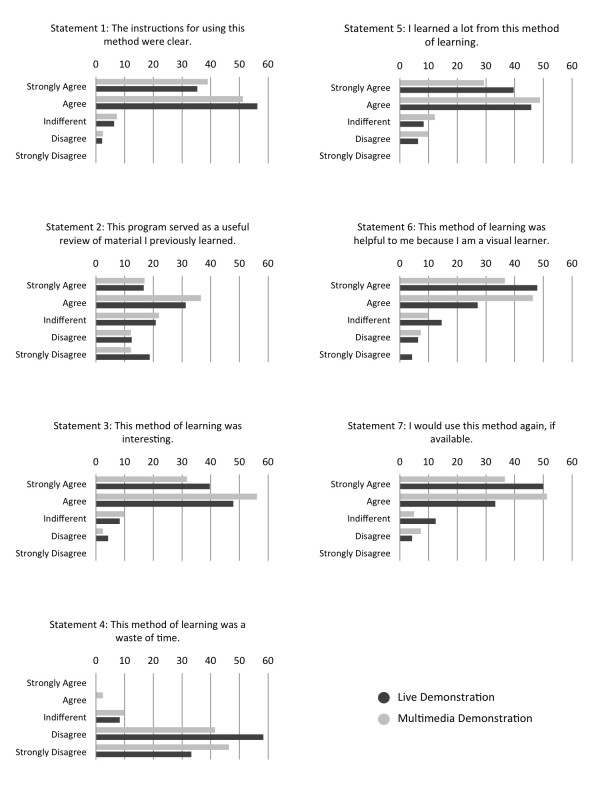
**Student responses to Likert scale statements**. Results of students' responses to several statements.

Mann-Whitney U compared student responses to knee and ankle Likert statements for the two instructional groups. No significant differences were found (Table 3). Response frequency for student attitudes toward instructional strategy for knee and ankle are presented in figure 2. Forty-one subjects from the combined knee and ankle multimedia groups participated in the survey, for a response rate of 82%. Forty-eight subjects for the combined knee and ankle live demonstration groups participated in the study, for a response rate of 96%. Of the 41 students in the multimedia group, 87.8% either agreed or strongly agreed, while 2.4% either disagreed or strongly disagreed that the "method of learning was interesting". Within the live demonstration group, 87.5% either agreed or strongly agreed that the "method of learning was interesting", while 4.2% either disagreed or strongly disagreed. The remaining students were neutral. When asked if the "method of learning was a waste of time", 87.8% of the students in the multimedia group either disagreed or strongly disagreed, while 2.4% agreed or strongly agreed, and 91.6% of the students in the live demonstration group disagreed or strongly disagreed, while 0% agreed or strongly agreed. Of the subjects in the multimedia group 78.1% of the students agreed or strongly agreed they "learned a lot from this method of learning", while 9.8% either agreed or disagreed. Within the live demonstration group, 85.4% agreed or strongly agreed they "learned a lot from the method of learning" and 6.3% either disagreed or strongly disagreed. When asked if they would "use the method again", 87.8% of the multimedia group agreed or strongly agreed, and 83.3% of the live demonstration group agreed or strongly agreed. Only 7.3% of the multimedia group and 4.2% of the live demonstration group disagreed or strongly disagreed.

**Table 3:** Mann-Whitney Test for Likert scale statements

Statement	Knee			Ankle		
	
	U(df)	Z	p	U(df)	Z	p
The instructions for using this method were clear.	262	-0.12	.990	216	-0.41	.684

This program served as a useful review of material I previously learned.	189	-1.67	.095	183	-1.21	.228

This method of learning was interesting.	244	-0.47	.641	186	-1.23	.221

This method of learning was a waste of time.	207	-1.39	.166	225	-0.15	.881

I learned a lot from this method of learning.	216	-1.12	.262	211	-0.51	.612

This method of learning was helpful to me because I am a visual learner.	243	-0.46	.643	191	-1.02	.310

I would use this method again, if available.	247	-0.39	.698	203	-0.73	.467

Compares CD-ROM and live demonstration instructional strategies.

*Significance ≤ .05.

### Qualitative Results

The open-ended questions on the survey allowed students to discuss, in their own words, their opinions on various aspects of the teaching methods employed in this study. Tables 4 and 5 list the questions and a summary of the responses students provided. Table 6 displays results of open-ended questions instructors answered at the end of the study.

**Table 4:** Open-ended responses for students receiving CD instruction

Question	Response (Response Frequency*)
What did you like best about the method of learning?	"I could replay the CD if needed" (19) "The video part"/"visuals" (7) "Could review on my own time" (7) "Good reference for later" (6) "Self paced" (4) "The audio descriptions of what was happening on the video" (4)

What did you like least about the method of learning?	"Nothing"/"no complaints" (8) "No one to answer questions while watching the video" (7) "Some videos were hard to see" (5) "Prefer to see it in person" (5) "Wasn't able to get immediate feedback on performance" (5)

What are the strengths of this method of learning?	"Videos always available to view" (9) "You can watch videos many times" (9) "Helpful for visual learners" (9) "Good for test preparation" (7) "Good reference for the future" (7) "Can learn at home", "can learn alone" (4)

What are the weaknesses of this method of learning?	"Cannot ask questions" (9) "No human contact" (8) "N/A" (7) "None", "no complaints" (6) "No immediate feedback" (4)

Compared to other ways of learning, was this method of learning useful? Why or why not?	Yes: 30 responses "Can be referenced any time" (8) "Can work at own pace" (7) "Visual aid/visual learner" (9) "Helpful for studying" (6) No: 5 responses Same: 2 response Other: 5 response "It would be more useful if both techniques were combined" (4)

Numbers in parenthesis represent the number of similar responses.

*Note: Only responses reported more than 3 times were included in the table.

**Table 5:** Open-ended Responses for Students Receiving Live Demonstration Instruction

Question	Response (Response Frequency*)
What did you like best about the method of learning?	"Instructor Feedback"/"Immediate feedback" (15) "More contact time with instructor" (8) "Real person demonstration" (7) "Hands-on experience" (6) "Three dimensional visuals" and "Easy to see" (5) "It was visual" (4)

What did you like least about the method of learning?	"Went through tests too fast" (6) "Not enough time" (6) "Wanted the CD for practice while studying (6) "Nothing" (4) "Not enough one-on-one time"/"Too many people in class" (4)

What are the strengths of this method of learning?	"Fast feedback from the instructor" (12) "Able to ask questions" (11) "Knowledge of teacher" (5) "Hands-on" (5) "Good for visual learners" (5)

What are the weaknesses of this method of learning?	"Limited practice time" (11) "No video or pictures for review" (11) "Can't go back to see again" (6) "No complaints" (5)

Compared to other ways of learning, was this method of learning useful? Why or why not?	Yes: 33 "Immediate instructor feedback" (6) "Hands-on" (4) No: 3

Numbers in parenthesis represent the number of similar responses.

*Note: Only responses reported more than 3 times were included in this table.

**Table 6:** Instructors' Evaluation of Instructional Method

	Response and/or Response Frequency	
**Question**	**CD Instruction**	**Live Demonstration Instruction**

What did you like best about this method of instruction?	**Program 1: **"Smaller group size"; "CD-ROM provided a framework for interaction with the students"; "My time spent improving on student's technique and providing additional clinical information rather than just a 'how to'" **Program 2: **"Students had the convenient access as a reference for study outside of the classroom"	**Program 1: **"There was control over the flow and pace of the class"; "I could keep them on track in completing the class objectives"; "Everyone was working on a single skill simultaneously, so that I could quickly check proficiency" **Program 2: **"I was able to explain, discuss and apply the material without a time limit on instruction as was present in the CD"

What did you like least about the method of instruction?	**Program 1: **"Some students will not participate even if you ask, they state that they have already practiced or that they are finished" **Program 2: **"Video portion was too short not allowing sufficient time to discuss the demonstration"	**Program 1: **"Students do not have good notes or consistent stored memories of how to complete the skill"; "I demonstrate things but students do something different compared to the demonstration" **Program 2: **"Nothing"

What are the strengths of this method of instruction?	**Program 1: **"Students who are self-starters get a lot out of it, they get right down to business and work through all of the skills"; "It allows students to work at their own pace"; "Provides permanent reference" **Program 2: **"Students that viewed the material before class would have viewed the realism of the demonstration"	**Program 2: **"Students follow a consistent pattern in their learning"; "Students are engaged and attentive to the directions and during practice" **Program 2: **"Personal attention to detail of psychomotor techniques"

What are the weaknesses of this method of instruction?	**Program 1: **"Students who are not self-starters do not even get as much as they would out of demonstration"; "Dependent on the students' level of motivation and input"; "Some students had not spent much time with the CD-ROM, so they had to spend time going through the CD-ROM before practicing" **Program 2: **"Some students did not seem as interested nor as concentrated in the class learning as those who did not have the video"; "Some students did not practice as long with the techniques as did the students who did not have the CD"	**Program 1: **"Assessing everyone's abilities in each skill as they progress"; "Assuring that the students have enough information and level of proficiency that they can continue practicing independent of the instructor" **Program 2: **"Lacks the post classroom carry over as a reference for out of class study"

Compared to other ways of instruction, was this method instruction useful? Why or why not?	**Program 1: **"I think that this was useful - especially for students who took advantage of the opportunity"; "For some students this could be a stand-alone tool, other students could use live demonstration and feedback as stand-alone"; "For many students, demonstration augmented with CD-ROM would be great" **Program 2: **"I did not like the limited time that the CD video clip had per psychomotor technique"; "Many teaching items were left out due to the time factor"	**Program 1: **"Absolutely; very useful"; "Supervised instruction is probably the best method to obtain a consistent level of performance"; "Feedback, individualized assistance and responding to individual variability is essential in performance development" **Program 2: **"Yes, see #1 above"

## Discussion

This study compared the effects of instructional multimedia and live demonstration of psychomotor skills on student study behavior and student/teacher attitudes. The first guiding research question was: "What are the attitudes of students and instructors using instructional multimedia as an instructional strategy?"

Attitudes were measured by a modified questionnaire used in Toth-Cohen's (1995) investigation of the effects of instructional multimedia on occupational therapy student understanding of anatomy and kinesiology. Toth-Cohen found more positive attitudes in quantitative and qualitative responses from students receiving instructional multimedia as compared to students receiving traditional self-study with textbooks [38]. In the present study, responses by students were similar regardless of method of instruction. The differences in results noted between the Toth-Cohen study and this study may relate to sample characteristics, differences in the type of skills taught, access to the instructional strategy, and/or differences between the instructional methods in the present study versus Toth-Cohen's study. Participants in Toth-Cohen's study were undergraduate occupational therapy students as compared to graduate physical therapy students in this study. Additionally, Toth-Cohen limited content to cognitive skills related to the elbow as compared to this study's emphasis on cognitive and psychomotor skills.

Student responses to the open-ended questions in this study were consistent with Toth-Cohen [38]. Strengths of the instructional multimedia presentation reported by students in the present study included greater opportunity to view and/or review content and control of the pacing and content quantity. Instructors also identified greater opportunity for review of the content as a major strength of CD presentation and highlighted the facilitation of self-directed learning and increased efficiency. Instructors reported observing higher cognitive level interactions during lab time with students in the interactive multimedia group. The students in our study identified weaknesses of instructional multimedia as including the lack of direct human contact and visual limitations of the CD. Instructors reported decreased student participation and decreased preparation by some participants in the instructional multimedia groups.

Conversely, students appreciated the hands-on aspect of live presentation but disliked the lack of detail, excessive speed of presentation, memory overload, and lack of resources for continued study of live presentation. Instructors perceived having greater control of the teaching-learning interaction and greater participant attention to the details of the techniques during live presentation. Predictably, students reported a greater sense of autonomy with the instructional multimedia presentation and the instructors reported a greater sense of autonomy with live presentation. Another contrasting view related to the detail of the instruction. Instructors reported greater detail with live presentation, however the students reported less detail with live presentation.

The degree of control that students are able to exercise in a course activity depends on the structure and flexibility designed into the activity by the instructor. Instructor satisfaction derives from congruence between the instructor's beliefs about learning and the methods the instructor uses to bring about learning, and most adult educators value high levels of student control and involvement [29]. If this tendency extends to physical therapy educators, then instructors should realize increased satisfaction when teaching a course in which students have opportunities to control their learning, as with instructional multimedia for practicing skills.

Benefits of instructional multimedia for self-directed learning include flexibility in time and place of learning and continual access to the learning materials. Technology used in support of unlimited student practice can be a central strategy for the type of mastery learning that supports adult learners' self-efficacy and persistence in education [39]. However, these benefits can be neutralized if students do not find the technology easy to use. Student attitudes toward using the technology and toward the value of learning new technology will impact their motivation and satisfaction to learn [40]. If students believe that their professional value is enhanced by acquiring technology skills, their motivation and satisfaction will increase.

The performance-driven benefit of student satisfaction is of particular importance because of its task, course, and program level implications. Students who are satisfied with their own performance and with their progress are more likely to persist in their educational endeavor [35]. Therefore, it is in the best interests of the student and instructor to reach a motivating level of satisfaction in their courses. In the case of the physical therapy courses investigated in this study, students were asked to use instructional multimedia for self-directed learning, a method for which students vary in their readiness and need for support [36]. When teaching a varying group of students, the instructor should expect a range of satisfaction outcomes unless differential amounts of self-direction are afforded, as is the case with interactive media. The continual need for learning within individuals and the need to explore and to develop responsibility among younger adult learners are well satisfied with multimedia tools that students can control and use as their abilities dictate [37].

The second guiding research question was "Does instructional multimedia affect student study behavior?" This study demonstrated no significant differences in study time with classmates, study time alone, or total study time between the instructional strategies for the knee. However, participants receiving instructional multimedia of ankle skills reported significantly less study time with classmates than participants receiving live presentation of the ankle content. No difference was observed for study time alone or group study time between the instructional strategies for the ankle. The decreased study time found with total study time with the ankle instructional multimedia group was consistent with the decrease in study time noted in physical therapy students using computer-assisted instruction (CAI) in the study by Kinney *et al*. (1997). The CAI group in the Kinney study completed a lesson related to examination and treatment of a patient with carpal tunnel syndrome in approximately 83 minutes as compared to 125 minutes for the lecture group [19]. The difference between the results of Kinney *et al*. and this study maybe related to student level and the operational definition of study time. The students participating in Kinney's study were undergraduate students. Kinney included class time as a component of study time as compared to only out-of-class study time measured in this study. The improved classroom efficiency of approximately 25% noted by the teachers in this study was consistent with the 24% efficiency reported by Kinney *et al*. (1997) [19].

### Future directions and limitations

Interestingly, while students reported an appreciation of the autonomy received with the use of the instructional multimedia, instructors reported an appreciation of the autonomy that they received from live presentation. This conflicting view of autonomy raises several pedagogical issues for future researchers. The level of perceived autonomy, not measured in this study, and the effect of this autonomy on student performance is of significant interest, particularly to educators of adult learners. The conflicting views between students and instructors of the level of detail provided by the instructional strategies in this study are of interest as well. Students attributed greater detail to the instructional multimedia as opposed to instructor perception of greater detail from their own presentations. These conflicts raise questions related to instructor pedagogy not assessed in this study. Future research of the role of instructor pedagogy in the application of interactive multimedia is indicated. The findings of this study are limited to written answers from students and teachers without follow-up clarification of their answers. Future investigations into these conflicting views and their effects are indicated and may include interview and other methods to clarify. Conflicting views between instructors and students of the benefits of instructional multimedia and live presentation were identified by the results of this study.

A limitation of this study is the inclusion of only two physical therapy programs. Future multi-site investigations are needed with attention to curricular implications. The use of a non-validated survey tool limits the generalizability of the findings. While used previously in the literature, the survey from Toth-Cohen (1995) has not been extensively examined for reliability and validity. Other limitations include student self-reporting of the study time.

Since the conclusion of the study the authors have started to distribute video using a university owned media server and through Apple's iTunes. Video being distributed now is in MPEG-4 format.

## Conclusions

This study investigated the effects of instructional multimedia on student/teacher attitudes and student study behavior. No practical differences between the instructional groups were noted between student attitudes toward the instructional method as measured by seven statements with Likert scales. Responses to five open-ended questions relative to instructional multimedia emphasized efficiency, processing level, autonomy, and detail of instruction of instructional multimedia. This study suggests that instructional multimedia may improve efficiency and may promote higher level processing during practice of the techniques in a supervised setting.

## Competing interests

The authors declare that they have no competing interests.

## Authors' contributions

ARS and CC conceived and designed the study. All authors collected and/or analyzed data. All authors were involved in writing and revising the original manuscript. WAM and ARS designed the tables and prepared the paper for submission. All authors read and approved the manuscript prior to submission.

## Acknowledgements

Publication of this article was funded in part by the University of Florida Open-Access Publishing Fund.

## Appendix 1 - Student Experience Questionnaire

EVALUATION OF EXPERIENCE WITH METHOD OF LEARNING

I. Please check the appropriate box below:

□ CD Group   □ Classroom Presentation

II. Please circle the correct answer below:

1. **The instructions for using this method were clear.**

Strongly disagree   Disagree   Indifferent   Agree   Strongly agree

0   1   2   3   4

2. **This program served as a useful review of material I previously learned.**

Strongly disagree   Disagree   Indifferent   Agree   Strongly agree

0   1   2   3   4

3. **This method of learning was interesting.**

Strongly disagree   Disagree   Indifferent   Agree   Strongly agree

0   1   2   3   4

4. **This method of learning was a waste of time.**

Strongly disagree   Disagree   Indifferent   Agree   Strongly agree

0   1   2   3   4

5. **I learned a lot from this method of learning.**

Strongly disagree   Disagree   Indifferent   Agree   Strongly agree

0   1   2   3   4

6. **This method of learning was helpful to me because I am a visual learner.**

Strongly disagree   Disagree   Indifferent   Agree   Strongly agree

0   1   2   3   4

7. **I would use this method again, if available.**

Strongly disagree   Disagree   Indifferent   Agree   Strongly agree

0   1   2   3   4

*III. Please answer the following questions*.

8. **What did you like best about the method of learning?**

9. **What did you like least about this method of learning?**

10. **What are the strengths of this method of learning?**

11. **What are the weaknesses of this method of learning?**

12. **Compared to other ways of learning, was this method of learning useful? Why or why not?**

13. **Any other comments?**

14. **Please estimate below the total number of minutes you studied this content *outside *of the classroom:**

alone___________________ minutes

with classmates___________________ minutes

## Appendix 2 - Instructor Experience Questionnaire

TEACHER EVALUATION OF INSTRUCTIONAL METHOD

I. Please check the appropriate box below:

■ CD Group   ■ Classroom Presentation

*II. Please answer the following questions relative to the method checked above*.

1. **What did you like best about this method of instruction?**

2. **What did you like least about this method of instruction?**

3. **What are the strengths of this method of instruction?**

4. **What are the weaknesses of this method of instruction?**

5. **Compared to other ways of instruction, was this method instruction useful? Why or why not?**

6. **Any other comments?**

## Pre-publication history

The pre-publication history for this paper can be accessed here:

http://www.biomedcentral.com/1472-6920/11/38/prepub
